# Book Notices

**Published:** 1855-04

**Authors:** 


### BOOK NOTICES.

*Fourth Annual Report of the Trustees of the Illinois State Hospital for the Insane.* Jacksonville: Dec. 1854.

*Reports of the Trustees and Superintendent of the Butler Hospital for the Insane*, presented at the Annual Meeting in January, 1855. Providence, R. I.

*Nineteenth Annual Report of the Managers of the New York Institution for the Blind*, to the Legislature of the State, made January, 1855. New York: James Egbert. Printer.

*Physician's Report on Closing the Roper Hospital after the Yellow Fever Epidemic of* 1854, made to the Trustees of the Institution. By W. T. WRAGG, M.D., Physician to the Hospital. Charleston, S. C. 1855.

*Sanitary Reports of the City of Buffalo for the Year* 1854 By the Board of Health. Buffalo, N. Y.: Clapp, Mathers & Co., Printers.

FEW things afford a more pleasing index of the humanizing tendencies of the present age, than the measures adopted for the care of the helpless, the unfortunate, and the sick.

The hospitals for the sick and insane, and the asylums for the blind and deaf, are so many monuments pointing to the triumphs of reason and benevolence over human ignorance and selfishness.

The first of the reports named above is a pamphlet of 51 pages, containing the Reports of the Trustees and Superintendent of the Hospital for the Insane, at Jacksonville, Illinois; and also the laws of the State regulating the admission of patients, &c.

From it we learn that the hospital is under the immediate charge of Andrew McFarland, M D., Superintendent; Charles C. Cornett, M.D., Assistant Physician; John Henry, Steward; Eliza M. Rogue, Matron; and Isabella Henry, Housekeeper. The report of the Trustees relates chiefly to the financial condition of the hospital, and contains nothing of special interest to our readers. From the report of the Superintendent we learn that: "At the date of the last report, there remained in the institution eighty-two patients. Since that time there have been admitted two hundred and sixty-five cases; making the whole number who have enjoyed the benefits of the hospital from December 1st, 1852, to December 1st, 1854, three hundred and forty-seven. During the same period nearly one hundred and eighty two have been discharged, leaving one hundred and sixty-six now under treatment. As the hospital affords accommodation for the two sexes equally, and the whole had been occupied the most of the time recently, the number of males and females has been nearly the same."

Of the 182 reported discharged, 21 were discharged by death, 16, by order of the Trustees, unrecovered, 31, by request of friends, unrecovered, and the remaining 114 recovered. These results compare favorably with those exhibited by the best institutions for the insane in our country, and speak well for the skill and faithfulness of those who have the institution in charge. There is one recommendation in the report of the Superintendent with which we should decidedly differ. It relates to, and encourages the plan of aggregating large numbers of the insane in one institution. On this subject we fully agree with Dr. L. V. Bell, and others who have had much experience in the care of the insane, that no one institution should contain at one time more than from 200 to 250 patients.

The second report named in the caption is from the Trustees and Superintendent of the Butler Hospital for the Insane at Providence, R. I. The institution was founded in 1848, and is under the immediate management of Isaac Ray, M.D., Superintendent and Physician; Roger C. Perkins, M.D., Assistant Physician; and Mrs. Sarah D. Lovett, Matron. Dr. Ray is well known among those who have devoted much attention to the insane, and his report will be read with interest. The following paragraph will show the number cared for in the institution during the year 1854, and the results of such care, viz.:

"On the 31st of December, 1853, there were in the house one hundred and thirty-six patients—sixty-three males and seventy-three females. During the year ending the 31st of December, 1854, there were admitted eighty—thirty-one males and forty-nine females; making two hundred and sixteen under care in the course of the year. There have been discharged eighty-five—forty males and forty-five females; leaving, on the 31st of December, 1854, one hundred and thirty-one patients. Of the persons admitted, twenty-two were from other States. Of the remaining fifty-eight, twenty one were supported by cities and towns, six partly by the bounty of the State, dispensed by the Governor, and thirty-one entirely by private means. Of those discharged, forty had recovered, twenty were improved, six were unimproved, and nineteen died."

The third report in the list named is that from the New York Institution for the Blind, which has now been in operation *nineteen* years. The special design of this noble institution is to afford instruction, both educational and mechanical, to the hopelessly blind. T. Colden Cooper is the Superintendent, aided by a full board of teachers—literary, musical, and mechanical. And we know of no public institution that more fully deserves the confidence of the people than this. The following paragraph will show the number receiving the benefits of the institution during the the year 1854, viz.:

"The whole number of blind persons thus receiving education, or the means of support, through the medium of the institute, is two hundred and five. Of the one hundred and forty-two pupils, one hundred and sixteen are beneficiaries of the State of New York, six are supported by the State of New Jersey, thirteen by their friends, two by the Commissioners of Emigration, and five are received gratuitously by the institution."

Only three deaths occurred among the inmates during the year. In addition to the usual topics treated in this report, is an interesting report, by J. G. Adams, M.D., one of the Board of managers, giving "an account of the Institutions for the Blind visited by him during his absence in Europe during the years 1853—4." The institutions spoken of by Dr. Adams are, the *Institut des*

*Jeunes Aveugles*, Paris; the *School of the Indigent Blind*, in St. George's Fields, Southwark, London; and the *School for the Blind*, in Hardman St., Liverpool.

We cannot analyze this paper without giving it entire; and for this we have not room at present. Dr. Adams states the proportion of blind persons in Europe to be 1 in 1400 inhabitants; in the United States, somewhat larger; while in Egypt it is as high as one in every 100.

The fourth report named in the list at the head of this paper is an exceedingly interesting one, from Dr. W. T. Wragg, of Charleston, S. C. The Roper Hospital seems to have been a temporary institution, made necessary by the severe prevalence of Yellow Fever in the city of Charleston during the summer and autumn of 1854. The report of Dr. Wragg contains very much interesting and valuable matter; but it is so judiciously condensed and statistical in its arrangement, that no adequate idea can be given of its contents by a further analysis. The following, in relation to the nature of Yellow Fever, and the conditions of the urine during its progress, is well worthy of careful consideration, viz:

And I may, perhaps, not be going beyond the sphere of my duty on this occasion, in suggesting a connection between this vitiated state of the fluids of the stomach and a diseased condition of the great sympathetic nerve, indicated by that symptom which was shown above to have been the only *invariable* attendant of the disease, viz:—Pain in the back. I am of opinion that if ever we arrive at such perfection in our means of examining the nervous system, as to be able to detect the existence of disease with certainty as well as accuracy in these tissues, we will find that in yellow fever the sympathetic system is in that state, and, as a consequence, deranges all the secretory actions of the digestive apparatus. Such a view enables us to account for all the symptoms. The deep seated pain along the dosal region, most violent at the points corresponding with the plexuses of the nerve in question, and the radiations of this pain from these points to the distributions of the nerve, in some cases involving the entire area of its ramifications; the intense nausea and obstinate vomiting, which are found to begin long before any traces can be discovered of lesion in any other of the tissues of the stomach; the entire alteration of all the secretions, and consequent arrest of the functions of digestion, &c. ; the tendency to early solution, and even dissolution of the blood; and, finally, the heavy, dull and painful oppression in the whole abdominal region, including the space from the umbilicus to the pubis, are strongly inferential, if not positive proofs of the diseased condition of this great system. By reflex action from this important nerve, the whole spino-cerebral system is brought into disordered action, and hence the train of general symptoms familiar to all.

This, however, is not the place to elaborate such views. I have referred to them as explanatory of other derangements of the general system, which I must now proceed to touch briefly upon.

Before leaving the intestinal canal and its disordered fnnctions, I will refer to one other appearance as corroborative of the ideas suggested in relation to the existence of muriatic acid. As the blue or ocean-green color of the vomited fluid is seemingly due to this acid, mixed with bile or saliva in the upper part of the canal, so the grass-green hue of the stools appears to depend on a mixture of bile, with the same acid in the parts of that tube below the stomach. It is easy for any one to satisfy himself of these effects from such mixtures by experiments out of the body.

A symptom which was noted as of fearful consequence, was suppression of urine. It was present temporarily in a large numbor of the cases during the early part of the first stage, and in 15 it was persistent, continuing to three days. Of the 15 thus affected, 12 died, and but 3 recovered. This condition must be carefully distinguishable from the retention of urine, which was also common even when it could not be referred to stranguary. The latter was easily overcome, the former never yielded.

It may be as well for me to finish what I wish to say on this subject. Though scanty in quantity, yet often but slightly altered in the early stage of the fever, it offered deviations from the healthy standard in the second and third stages of great importance.

First: It was found to eliminate from the system an immense quantity of bile in those cases in which jaundice succeeded as one of the secondary symptoms.

Second: It carried off free hydrochloric acid in a considerable number of cases, as was shown when the tests for that substance were used; and I am disposed to believe, from the frequent presence of this acid in the stomach and intestines during the early stage of the fever, that its existence in the urine takes place earlier, and is more frequent than we are yet aware of. It has not been possible for me to institute a comparison between the number of deaths and recoveries in cases where this state of the urine was made out. It appears to have been coincident with great danger to the patient; but it will be a point of much interest to be investigated hereafter, whether its presence in the urine at this stage of the disease is not, as in the case of bile under the same circumstances, proof of the existence of an eliminative action.

Third: There were, in a few cases, red sedimentary deposits, consisting of urate of ammonia, colored with purpurine.

Fourth: Large quantities of organic debris, apparently epithelial scales, &c., were found in a few cases.

For these results of chemical analysis of the urine, I am indebted to Dr. Ford, whose careful researches, though not sufficiently corroborated by repetition, will, I am disposed to believe, when completed and given to the public, be found to open some new views of this disease."

The whole number of cases treated in the hospital was 254, of whom 92 died, and 162 recovered. Of the whole number, the *black vomit* occurred in 74. Of this number, 9 recovered after throwing it up, and 63 died. In relation to treatment, Dr. Wragg says: "I repeat that no reliance was placed on any specific, and though I tried all that I conscientiously thought that I might use with safety, or a hope of success, my chief dependence was on the application of such general principles as seemed to find their required conditions in the symptoms before me."

The fifth and last paper in the list given above is chiefly occupied with the report of James M. Newman, M.D., Health Physician, in relation to the *cholera* in the city of Buffalo, during the year 1854. The report occupies 43 pages, and is accompanied by Meteorological Tables, a chart showing the streets in which the cholera prevailed, and tables exhibiting the entire mortality of the city from all diseases. It is printed in good style, by order of the Common Council of the City. We are not sure but the City Physician and Common Council of Chicago might take a useful hint in relation to their own duties from this report. It is well drawn up, minute in its details, and well worthy of perusal. The appended tables show a gross mortality during the year, from all diseases, of 2,936: of which 572 are reported from epidemic cholera; 187 from cholera infantum; 21 from cholera morbus; 90 from dysentery; 266 from consumption; and from intemperance and delirium tremens 32.

The population of Buffalo is reported to be 80,000, which would make the average mortality of the city for 1854 about 1 in 27.

*On Injection of the Bronchial Tubes, and Tubercular Cavities of the Lungs.* By HORACE GREEN, M.D., LL.D., President of the Faculty, and Professor of the Theory and Practice of Medicine in the New York Medical College; Corresponding Fellow of the London Medical Society; Member of the American Medical Association, &c.

THIS is a monograph of 20 pages, re-published from the January number of the American Medical Monthly. In it the author not only defends his claim to priority in the practice of introducing a sponge probang into the larynx, for the treatment of laryngeal diseases but he claims to have recently extended this practice so far as to introduce a tube through the larynx into the bronchial tube, and inject solutions of nitrate of silver into the branches of the bronchia, &c. Dr. Green claims to have treated several cases by injecting from one to two drachms of a strong solution of nitrate of silver into the bronchial tubes, and with much benefit to the patients The following is the concluding paragraph of his paper:

"In conclusion, it is maintained that the direct medication of the lungs, by means of *catheterism of the air-tubes*, an operation I believe not before performed, has been repeatedly accomplished; that the operation may be performed by the dexterous surgeon with ease and facility, and with *perfect safety* to the patient; and that the results of this method of treating disease, whether it has been employed in bronchial affections, or in the commencement of tuberculosis, have already afforded the most gratifying indications that practical medicine will be greatly advanced by this discovery." *Mirabile dictu.*

---

*A Manual of Pathological Anatomy.* By C. HANFIELD JONES, M.B., F.R.S., Fellow of the Royal College of Physicians, Assistant Physician to, and Lecturer on Pathology at, St. Mary's Hospital; and EDWARD H. SIEVEKING, M.D., Fellow of the Royal College of Physicians, Assistant Physician to, and Lecturer on Materia Medica at, St. Mary's Hospital. First American Edition revised, with 397 Illustrations. Philadelphia. Blanchard and Lea. 1854.

THIS is the title to a large-sized octavo volume, well bound in calf, containing 733 pages. It is designed for a standard work or text-book on the very interesting and important subject of Pathological Anatomy. The following are the general topics discussed in the work, as indicated in the table of contents, viz: 1st. General Pathological Anatomy. 2d. Pathological Anatomy of the Alimentary Canal. 3d. Pathological Anatomy of the Joints. 4th. Pathological Anatomy of the Nervous System. 5th. Pathological Anatomy of the Organs of Circulation. 6th. Pathological Anatomy of the Organs of Respiration. 7th. Pathological Anatomy of the Female Organs of Generation. 8th. Pathological Anatomy of the Osseous System. 9th. Pathological Anatomy of the Urinary Apparatus. The chapters included under the three first and the last heads, are written by Dr. Jones, and those embraced under the other five divisions, by Dr. Sieveking. The American publishers have added many illustrations not contained in the London edition, and have also introduced into the chapter on cancerous growths the microscopical observations of Dr. Donaldson, of Baltimore, on the characteristics of the true cancer cell. We have not yet had time to examine the contents of the volume with that care which is necessary to enable us to make a critical analysis of its facts and doctrines; but we have no hesitation in recommending it as worthy of careful and thorough study by every member of the profession, whether old or young.

---

*Transactions of the New Hampshire Medical Society*, held at Concord, June, 1854.

THIS is a well-printed pamphlet of 56 pages, containing the proceedings of the State Medical Society during its *Sixty fourth Anniversary* meeting; the Annual Address of the President. Dr. Albert Smith, Professor of Materia Medica in Dartmonth College, N. H.; the Poetry of the Medical Profession, an Oration by Dr. Andrew McFarland, Superintendent of the Illinois State Hospital for the Insane; and a Dissertation on the necessity of a knowledge of the chemical changes that take place in the human body while in a state of disease, by Dr. W. H. H. Mason. These are well written productions. That of Dr. Smith is headed, "Conservatism in Medicine." And though interesting and well written. it is chiefly occupied with those general considerations in relation to the condition of the profession, and the various forms of quackery, which have constituted the staple of so many addresses during the last few years. We are almost tired of this species of literature, and could wish that every physician in the United States, instead of mentioning the name of any species of quackery, pathy, or ism. during the next five years, would bend all his energies to a thorough improvement of himself and the profession to which he belongs

The second paper is in the form of an Oration, by Dr. McFarland. now Superintendent of the Hospital for the Insane at Jacksonville, in this State. It occupies twelve pages, and as a literary production is highly creditable to its author. But it must be read entire to be appreciated. The paper of Dr. Mason occupies nine pages, and is a just appeal to the members of the Society in favor of a more thorough knowledge of the chemistry of man. We think a society *sixty four* years old, like that of New Hampshire, should present in its printed transactions more strictly scientific matter than is to be found in the pamphlet before us We fear our brethren in the Granite State need a little waking up.

